# Serum Amyloid A3 Expression Is Enhanced by Gram-Negative Bacterial Stimuli in Bovine Endometrial Epithelial Cells

**DOI:** 10.3390/pathogens14080729

**Published:** 2025-07-23

**Authors:** Kazuha Aoyagi, Keishi Owaki, Hiroki Sakai, Ayaka Okada, Yasuo Inoshima

**Affiliations:** 1 Laboratory of Food and Environmental Hygiene, Joint Department of Veterinary Medicine, Gifu University, 1-1 Yanagido, Gifu 501-1193, Japan; 2 Laboratory of Veterinary Pathology, Joint Department of Veterinary Medicine, Gifu University, 1-1 Yanagido, Gifu 501-1193, Japan; 3 Joint Graduate School of Veterinary Sciences, Gifu University, 1-1 Yanagido, Gifu 501-1193, Japan; 4 Education and Research Center for Food Animal Health, Gifu University (GeFAH), 1-1 Yanagido, Gifu 501-1193, Japan; 5 Center for One Medicine Innovative Translational Research (COMIT), Gifu University, 1-1 Yanagido, Gifu 501-1193, Japan

**Keywords:** lipopolysaccharide, lipoteichoic acid, bovine endometrial epithelial cells, serum amyloid A1, serum amyloid A3

## Abstract

Bovine endometritis is a common postpartum disease that significantly impairs reproductive performance and reduces economic sustainability in dairy and beef cattle. It is primarily caused by gram-negative and -positive bacteria, triggering strong inflammatory responses in the endometrium. Serum amyloid A (SAA) is an acute-phase protein and precursor of amyloid A (AA) in AA amyloidosis. In cattle, multiple SAA isoforms have been identified; however, the biological functions of SAA3 remain unclear. Hence, this study investigated the role of SAA3 in bovine endometrial epithelial cells (BEnEpCs) following stimulation with gram-negative or -positive bacterial antigens. BEnEpCs were treated with lipopolysaccharide (LPS) and lipoteichoic acid (LTA) and, subsequently, the expression levels of SAA3 and SAA1 mRNA were compared by real-time PCR. To further investigate protein-level changes, immunocytochemistry (ICC) was performed to assess the expressions of SAA3 and SAA1. These analyses revealed that SAA3 mRNA expression was significantly enhanced by LPS and LTA, whereas SAA1 mRNA remained undetectable or showed only minimal responsiveness. Notably, only SAA3 protein expression increased in response to stimulation. These results indicate that SAA3 plays a crucial role in the innate immune response of BEnEpCs against gram-negative bacteria. Our *in vitro* findings may facilitate understanding of the innate immune activity in bovine uterus.

## 1. Introduction

Endometritis is a common postpartum disorder in cattle, caused by bacterial infections and characterized by inflammation of the endometrial uterine lining [1,2]. It is a significant cause of reproductive inefficiency in both dairy and beef cattle, causing reduced conception rates, prolonged calving intervals, reduced milk yield, and increased culling rates [1,2,3]. Whereas the disease may be less frequently monitored in beef cattle due to differences in herd management, its effect on reproductive performance and economic sustainability is catastrophic [4]. Bovine endometritis pathogenesis involves several gram-negative and gram-positive bacteria, such as *Escherichia coli* and *Trueperella pyogenes*, being frequently isolated from the infected uterus [5,6]. These pathogens disrupt the normal postpartum uterine repair processes and elicit strong inflammatory responses that compromise fertility. The condition is worsened by subclinical infections, which are difficult to detect but significantly impair reproductive outcomes [7].

The innate immune system of the bovine mucosal epithelial cells is essential for recognizing and responding to microbial invasion. Among the innate defense molecules, serum amyloid A3 (SAA3) is upregulated in response to bacterial infection and may contribute to mucosal immune defense [8,9]. SAA is an acute-phase protein and precursor of amyloid A (AA) in the AA amyloidosis that occurs in response to chronic infections or chronic inflammatory disorders, such as rheumatoid arthritis and juvenile inflammatory arthritis [10]. Additionally, SAA is regarded as a major acute-phase protein in the plasma of various animal species, including humans, and is recognized as a diagnostic marker for infection and inflammation [11]. Based on differences in amino acid sequences, multiple SAA isoforms—SAA1, 2, 3, and 4—have been identified in several mammalian species, including humans, mice, and rabbits [12]. SAA1 and SAA2 are predominantly produced in the liver and are the primary circulating isoforms in the plasma [12]. The concentration of SAAs in plasma, primarily SAA1, significantly increases up to 1000-fold during inflammation [12]. In contrast, SAA3 is primarily expressed in extrahepatic tissues, such as the intestine and mammary glands [13,14]. In mice, SAA3 expression increases on the colonic surface in the presence of microbiota [15]. In a previous *in vitro* study, lipopolysaccharide (LPS) treatments strongly induced SAA3 mRNA expression in mouse colonic epithelial CMT-93 cells [15]. LPS is a membranous antigen of gram-negative bacteria, such as *E. coli*. Additionally, treatment of tumor necrosis factor α (TNFα), a pro-inflammatory cytokine that is responsible to LPS, induced SAA3 expression in adipocytes [16] and CMT-93 cells [15]. Moreover, systemic administration of LPS to mice *in vivo* induced SAA3 expression in the brain [17]. In bovines, SAA isoforms have been identified, with bovine SAA3 being differentially regulated based on the expression site [18]. LPS stimulation increases SAA3 expression in bovine mammary epithelial cells *in vitro* [18]. Moreover, both SAA3 mRNA and protein have been detected in the intestine, mammary glands, lungs, and uterus *in vivo* [19,20]. However, SAA3 expression and its biological function in the endometrial epithelia of the uterus remain unclear. Therefore, to facilitate understanding of the role of SAA3 in innate immune activity in the bovine uterus, SAA3 and SAA1 expression levels were compared in bovine endometrial epithelial cells (BEnEpCs) *in vitro* by stimulation with LPS and lipopolysaccharide (LTA), which are the membranous antigens of the major endometritis-causing gram-negative and -positive bacteria, respectively. We performed real-time PCR to measure SAA3 and SAA1 in BEnEpCs after treatments with LPS and LTA. Moreover, the protein expressions of SAA3 and SAA1 were analyzed using immunocytochemistry (ICC).

## 2. Materials and Methods

### 2.1. Cells

BEnEpCs were purchased from Cell Applications, Inc. (B932-05; San Diego, CA, USA) and cultured in BEnEpC basal medium (B910-400; Cell Applications Inc.) supplemented with 20% growth supplement (B911-GS; Cell Applications Inc.) in a 10 cm collagen-coated dish (NCO430167; Corning, Corning, NY, USA). Madin−Darby bovine kidney (MDBK) cells were cultured in Dulbecco’s modified Eagle’s minimal essential medium (044-29765, Wako, Osaka, Japan) with 5% fetal bovine serum (PAA Laboratories, Pasching, Austria), 100 U/mL penicillin, and 100 μg/mL streptomycin (15140-122, Gibco, Grand Island, NY, USA).

### 2.2. Treatment with LPS and LTA for mRNA Expression Analysis

Cells were seeded at a density of 3 × 10^5^ cells/well in six-well collagen-coated plates (4810-010N; IWAKI, Shizuoka, Japan) and incubated for 24 h at 37 °C in a 5% CO_2_ atmosphere. After rinsing the cells with phosphate-buffered saline (PBS; 045-29795; Wako), the cells were treated with LPS (L2630; Sigma-Aldrich, St. Louis, MO, USA) or LTA (L3265, Sigma-Aldrich), which are outer membrane proteins of gram-negative bacteria, *E. coli* O111:B4, or gram-positive bacteria, *Bacillus subtilis*, respectively. LPS and LTA were diluted to 0, 0.01, 0.1, 1, 10, and 100 µg/mL in BEnEpC basal medium without growth supplements. BEnEpC treated with LPS or LTA were incubated for 4 h at 37 °C for mRNA expression analysis.

### 2.3. RNA Extraction and cDNA Synthesis

RNA was extracted from LPS- or LTA-treated cells using the RNeasy Mini Kit (74106; Qiagen, Hilden, Germany) following the manufacturer’s protocol, and the RNA concentration was quantified using a NanoDropLite spectrophotometer (Thermo Fisher Scientific, Wilmington, DE, USA). DNA contamination in the extracted RNA was eliminated by treatment with DNaseI (18068-015; Invitrogen, Carlsbad, CA, USA), and cDNA was synthesized using PrimeScript RT Master Mix (RR036A; Takara, Kusatsu, Japan).

### 2.4. Cloning and Sequencing

Cloning was performed to obtain plasmid clones containing target genes to generate standard curves for absolute quantitative real-time PCR (qPCR).

PCR was performed on a Veriti thermal cycler (Applied Biosystems, Foster City, CA, USA) using GoTaq Hot Start Green Master Mix (M7122; Promega, Madison, WI, USA). SAA-specific PCR primers were designed (SAA1 mRNA F, 5′-GATCAGCACAATGAAGCTCTTCACAGGGCC-3′; SAA3 mRNA F, 5′-CACGGCCACAGGATGAACCTTTCCACGGGCA-3′; and SAA1,3 mRNA R, 5′-GAGAGGCAGCTCAGTACTTCTCAGGC-3′) based on bovine SAA1 (GenBank accession no. BC109787) and SAA3 (NM181016) sequences and used to amplify bovine SAA1 and SAA3 genes from the cDNA. Thermal cycling was performed for 2 min at 95 °C, followed by 40 cycles at 95 °C for 30 s, 55 °C for 30 s, and 72 °C for 30 s. The PCR products were purified using the QIAquick PCR Purification Kit (28106; Qiagen, Valencia, CA, USA) and ligated into the plasmid pTAC-2 using a DynaExpress TA PCR cloning kit (DS126L; BioDynamics Laboratory, Tokyo, Japan), following the manufacturer’s protocol. The recombinant plasmids were transformed into ECOS competent *E. coli* DH5α (310-06236; NipponGene, Toyama, Japan) following the manufacturer’s protocol. The recombinant bacterial colonies were selected on Luria–Bertani plates with 100 µg/mL ampicillin and confirmed through colony PCR using M13 primers (BioDynamics Laboratory). The PCR products were purified using a QIAquick PCR Purification Kit before their nucleotide sequences were determined by direct sequencing using a BigDye Terminator v3.1 cycle sequencing Kit (4337449; Applied Biosystems). Sequence analysis was performed using the Genetyx-Win software version 13 (Genetyx, Tokyo, Japan). Plasmids positive for amplicons were extracted using the QIAprep Spin MiniPrep Kit (27106; Qiagen) following the manufacturer’s protocol to generate standard curves in absolute qPCR.

### 2.5. Determination of Linear Range Using Plasmid-Derived Gene Standards

The plasmid concentration was measured using a NanoDropLite spectrophotometer (Thermo Fisher Scientific), and the plasmid gene copy number was calculated using the following equation:

(X × [6.0221 × 10^23^ molecules/mol])/([Y × 660 g/mol] × [1 × 10^9^ ng/g])

where X and Y represent the amplicon quantity in nanograms and nucleotide length of the plasmid with its specific target insert, respectively. Each plasmid contained only one amplicon, thereby acting as a proxy for the gene copy number. To establish a linear detection range, the plasmids were initially diluted to a stock concentration of 1 × 10^10^ copies/µL and, subsequently, serially diluted 10-fold to a lower value of 1 × 10^1^ copies/µL.

### 2.6. Absolute Quantification in qPCR

To measure the mRNA expression levels in cells, qPCR was performed in 96-well plates using 300 nmol each of forward and reverse primers, 10 ng of synthesized cDNA, and PowerUp SYBR Green Master Mix (A25742; Applied Biosystems) on a QuantStudio 3 real-time PCR system (Applied Biosystems). Thermal cycling was conducted for 2 min at 50 °C and 2 min at 95 °C, followed by 40 cycles at 95 °C for 3 s and 60 °C for 30 s. Specific primers were used to assess the mRNA expression levels of SAA1 (5′-AGTCCACAGCCAGTGGATGT-3′ and 5′-ATCTCTGAATATTTTCTCTGGCATC-3′) [19] and SAA3 (5′-CCTCAAGGAAGCTGGTCAAG-3′ and 5′-TACCTGGTCCCTGGTCATAC-3′) [21]. The SAA1 and SAA3 plasmid dilutions described above were used to generate standard curves to determine the absolute gene copy numbers of SAA1 and SAA3 mRNA in LPS- or LTA-treated cells. The cycle threshold values of the samples were compared with their respective standard curves to interpolate the gene copy number for each sample. The mean gene copy number with the standard error of the mean was plotted for each gene region under each condition. All experiments were independently repeated at least thrice.

### 2.7. Standard Curves for Measuring SAA1 and SAA3 mRNA Copy Numbers Through qPCR

**Figure A1:**
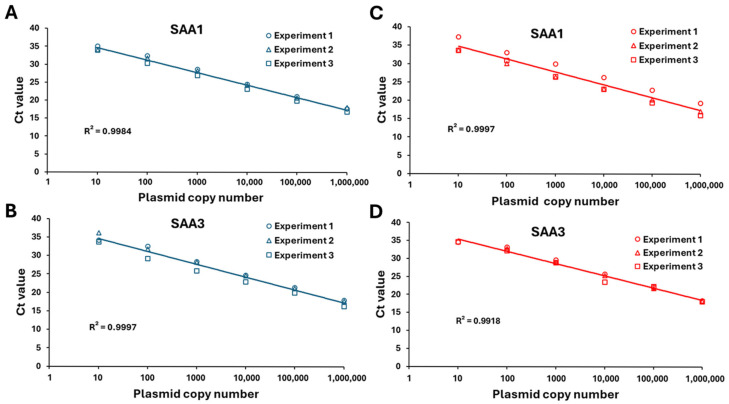
Standard curves for measuring SAA1 and SAA3 mRNA copy numbers in BEnEpCs treated with LPS (**A**,**B**) or LTA (**C**,**D**) using quantitative real-time PCR. SAA1 and SAA3 plasmid stocks are serially diluted 10-fold from 1 × 10^6^ to 10 copies to establish the linearity of amplification and detection range. To measure the within-assay variability, all reactions are performed in triplicate. Copy numbers are plotted against cycle threshold (Ct) values for SAA1 and SAA3 to determine R^2^ values. BEnEpCs, bovine endometrial epithelial cells; SAA, serum amyloid A; LPS, lipopolysaccharide; LTA, lipoteichoic acid.

Standard curves for qPCR were constructed using 10-fold serial dilutions (1 × 10^6^–1 × 10^1^ copies/µL) of SAA1 and SAA3 plasmid stocks to confirm the linearity of amplification and detection ranges. The copy numbers of SAA1 and SAA3 mRNA in BEnEpCs induced by LPS and LTA were determined based on these standard curves (Figure A1).

### 2.8. Treatment with LPS and LTA for ICC

BEnEpCs were seeded at a density of 1.5 × 10^5^ cells/well in eight-well collagen-coated chamber slides (SCS-N38; Matsunami Glass, Kishiwada, Japan) and incubated for 24 h at 37 °C in a 5% CO_2_ atmosphere. After rinsing the cells with PBS, the cells were treated with LPS or LTA. BEnEpCs treated with 0, 0.01, 1, and 100 µg/mL LPS or LTA were incubated for 4 h at 37 °C for ICC. MDBK cells treated with 100 µg/mL LPS were used as positive controls for SAA1.

### 2.9. ICC

ICC was performed to assess the SAA1 and SAA3 protein expression levels in LPS- and LTA-stimulated cells. LPS- or LTA-treated cells were washed with PBS and fixed with 4% paraformaldehyde for 20 min at room temperature and 100% methanol for 20 min at −20 °C. After washing with PBS, endogenous peroxidase activity was inhibited using 3% hydrogen peroxide for 20 min at room temperature and blocked non-specific antibody binding with 1% bovine serum albumin (BSA) in PBS for 30 min at room temperature. Anti-SAA1 monoclonal antibody 25BF12 (1:2000) [22] or anti-SAA3 monoclonal antibody 231G7 (1:1000) [19] were used as primary antibodies. The cells were incubated overnight at 4 °C with the antibody diluted in PBS containing 1% BSA. The following day, the cells were washed with PBS and incubated with horseradish peroxidase-conjugated anti-mouse IgG polyclonal secondary antibody (414311; Nichirei Biosciences Inc., Kyozan, Japan) for 30 min at room temperature. The cells were then washed with PBS, incubated with peroxidase in the ImmPACT DAB Substrate Kit (SK-4105; Vector Laboratories, Newark, CA, USA), and the wells were stripped from the slides. After counterstaining the nuclei with hematoxylin, the slides were dried and mounted using Mount Quick (DM01; DhythSangyo, Toda, Japan).

### 2.10. Statistical Analysis

Data were collected from at least three independent experiments, expressed as means ± standard deviations and analyzed for statistical significance using one-way analysis of variance and Bonferroni’s *post hoc* analysis.

## 3. Results

### 3.1. mRNA Expressions of SAA1 and SAA3 in BEnEpCs Treated with LPS and LTA

**Figure 1:**
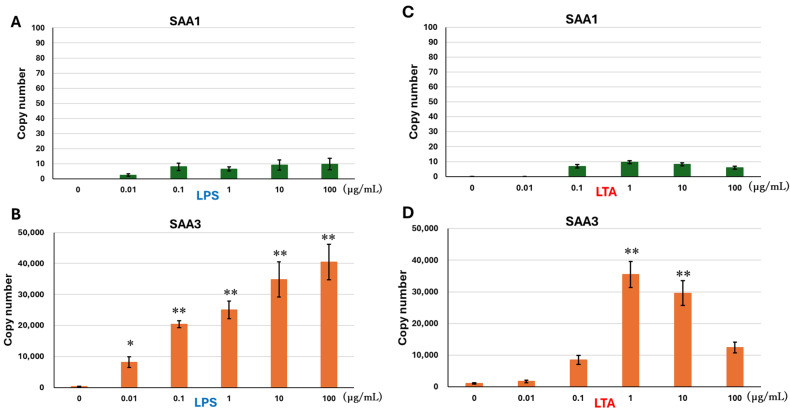
mRNA expressions of SAA1 and SAA3 in BEnEpCs treated with LPS and LTA. BEnEpCs were treated with 0–100 µg/mL of LPS (**A**,**B**) or LTA (**C**,**D**) at 37 °C for 4 h. mRNA expressions of SAA1 (**A**,**C**) and SAA3 (**B**,**D**) are determined by copy number using the standard curve (Figure A1). Data are represented as the mean ± standard deviation from three independent experiments. * *p* < 0.05 and ** *p* < 0.01. SAA, serum amyloid A; BEnEpCs, bovine endometrial epithelial cells; LPS, lipopolysaccharide; LTA, lipoteichoic acid.

SAA1 mRNA expression was not detected in the controls but became detectable following LPS treatment without showing a concentration-dependent increase (Figure 1A). Conversely, LPS treatment enhanced SAA3 mRNA expression in a concentration-dependent manner (Figure 1B), with significant upregulation at 0.1, 1, 10, and 100 µg/mL, peaking at 100 µg/mL.

Similar to the LPS treatment, SAA1 mRNA expression was not detected in the controls but became detectable following LTA treatment without showing a concentration-dependent increase (Figure 1C). LTA treatment enhanced SAA3 mRNA expression (Figure 1D). LTA at 1 and 10 µg/mL significantly stimulated SAA3 mRNA expression, with the greatest enhancement observed at 1 µg/mL. SAA3 mRNA expression was higher in the LPS-treated cells than that in the LTA-treated cells, excluding 1 µg/mL (Figure 1B,D).

### 3.2. Protein Expressions of SAA1 and SAA3 in BEnEpCs Treated with LPS and LTA

**Figure 2:**
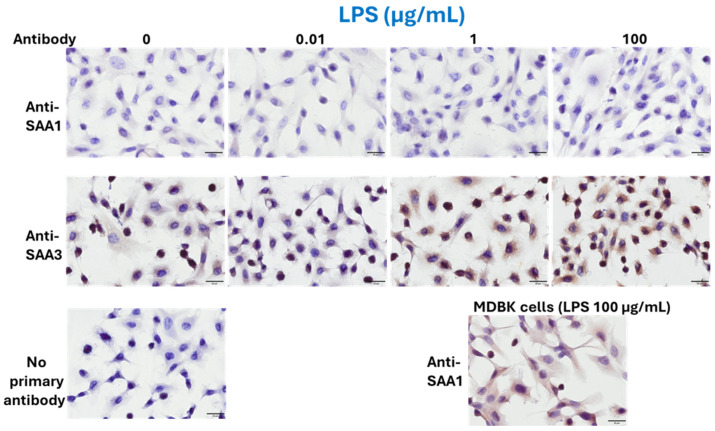
Protein expressions of SAA1 and SAA3 in BEnEpCs treated with LPS. BEnEpCs were treated with 0, 0.01, 1, and 100 µg/mL of LPS at 37 °C for 4 h and stained with anti-SAA1 or anti-SAA3 monoclonal antibodies, 25BF12 or 231G7. Cells are minimally stained with anti-SAA1 antibody with or without LPS treatment. Conversely, cells are strongly stained with anti-SAA3 antibody at 1 or 100 µg/mL of LPS. Madin–Darby bovine kidney (MDBK) cells were used for positive control of the staining of anti-SAA1 antibody. Scale bar = 20 µmm. BEnEpCs, bovine endometrial epithelial cells; SAA, serum amyloid A; LPS, lipopolysaccharide.

According to ICC results, BEnEpCs were minimally stained with an anti-SAA1 monoclonal antibody, regardless of LPS treatment, whereas LPS-treated MDBK cells were strongly stained (Figure 2). In contrast, BEnEpCs were strongly stained with the anti-SAA3 monoclonal antibody at 1 and 100 µg/mL LPS treatment (Figure 2).

**Figure 3:**
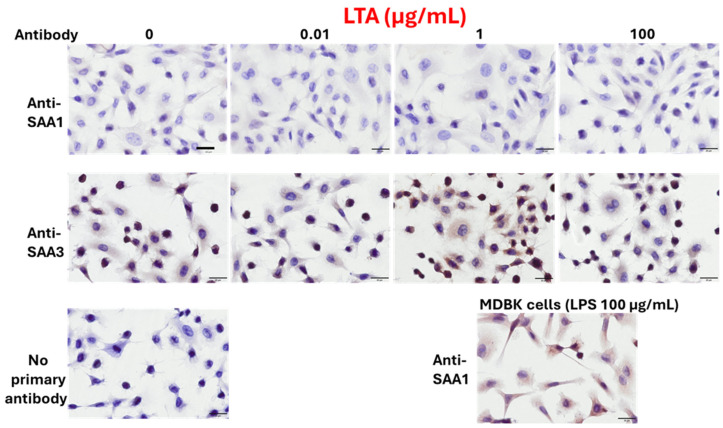
Protein expressions of SAA1 and SAA3 in BEnEpCs treated with LTA. BEnEpCs were treated with 0, 0.01, 1, and 100 µg/mL of LTA at 37 °C for 4 h and stained with anti-SAA1 or anti-SAA3 monoclonal antibodies, 25BF12 or 231G7. Similar to LPS treatment, cells are minimally stained with anti-SAA1 antibody with or without LTA treatment. Conversely, cells are strongly stained with anti-SAA3 antibody at 1 µg/mL of LTA. Madin–Darby bovine kidney (MDBK) cells were used for positive control of the staining of anti-SAA1 antibody. Scale bar = 20 µm. BEnEpCs, bovine endometrial epithelial cells; SAA, serum amyloid A; LTA, lipoteichoic acid; LPS, lipopolysaccharide.

BEnEpCs were minimally stained with the anti-SAA1 monoclonal antibody, regardless of LTA treatment (Figure 3). In contrast, BEnEpCs were strongly stained with anti-SAA3 monoclonal antibody at 1 µg/mL LTA treatment and weakly stained at a higher concentration (100 µg/mL). The dose-dependent expressions of SAA1 and SAA3 proteins in BEnEpCs were consistent with their mRNA expression patterns.

## 4. Discussion

Previously, we showed that bovine SAA3 mRNA expression was dose-dependently upregulated by LPS in bovine small intestinal epithelial cells and mammary epithelial cells, wherein SAA1/2 mRNA expression was unaffected [19]. Therefore, we hypothesized that LPS may induce SAA3 mRNA expression in other bovine epithelial cells. Herein, LPS stimulation induced SAA3 mRNA expression levels in BEnEpCs but had only a slight effect on SAA1 mRNA expression. Kiku et al. [23] analyzed gene expression profiles in primary bovine mammary epithelial cells after LTA stimulation and revealed that SAA3 was not among the top 20 regulated genes. In this study, SAA3 mRNA expression in bovine epithelial cells increased in response to LTA stimulation. However, its expression was lower than that observed in LPS-stimulated cells. The results of this study and those of a previous report indicate that SAA3 mRNA expression is predominantly induced by gram-negative bacterial infection [23].

The dose-dependent increase in protein expression was consistent with the mRNA expression results following LPS and LTA stimulation, indicating that the expressed SAA3 mRNA was translated into protein without inhibitory effect. In contrast, SAA1 protein levels were not increased by LPS and LTA stimulation, consistent with the mRNA expression results.

As the expression levels of inflammatory cytokines such as interleukin (IL)-6, IL-8, and tumor necrosis factor-alpha are significantly increased in BEnEpCs in response to *E. coli* stimulation [24], SAA3 may also be involved in defense against bacterial infection in the cattle uterus, alongside these cytokines. Transcriptome analysis by Zhou et al. [25] demonstrated that 36 genes were upregulated in bovine endometrial epithelial cells after LPS stimulation, in which SAA3 mRNA was significantly increased. A previous study showed that recombinant caprine SAA3 treatment reduced the infection rate of mastitis-causing bacteria in primary bovine mammary epithelial cells *in vitro* [26]. Therefore, SAA3 would play a role in activating innate immunity against bacterial infections in bovine epithelia, together with various cytokines. Bacteria in the uterus of cattle are typically eliminated within 1–3 months after calving; however, cattle with reproductive issues retain uterine bacteria for a prolonged period [27]; compromised uterine defense mechanisms result in post-calving infections [28]. To confirm the relationship between SAA3 and the host’s innate immunity, we must assess whether SAA3 stimulates the expression of genes involved in innate immunity, both *in vitro* and *in vivo*.

In summary, the results of this study indicate that SAA3 mRNA and protein are expressed in bovine endometrial epithelial cells due to bacterial infections, being particularly sensitive to gram-negative bacteria. These *in vitro* results suggest that similar responses may occur *in vivo* and are involved in innate immunity to bacterial infections.

## Data Availability

The data presented in this study are available in the article and Appendix A.

## Abbreviations

The following abbreviations are used in this manuscript:

AA	Amyloid A
BEnEpC	Bovine endometrial epithelial cell
BME	Bovine mammary epithelial
BSA	Bovine serum albumin
ICC	Immunocytochemistry
IL	Interleukin
LPS	Lipopolysaccharide
LTA	Lipoteichoic acid
MDBK	Madin–Darby bovine kidney
MUC2	Mucin2
PBS	Phosphate-buffered saline
PCR	Polymerase chain reaction
qPCR	Quantitative real-time polymerase chain reaction
SAA	Serum amyloid A
